# Real-time 3T MRI-guided cardiovascular catheterization in a porcine model using a glass-fiber epoxy-based guidewire

**DOI:** 10.1371/journal.pone.0229711

**Published:** 2020-02-26

**Authors:** Xinzhou Li, Luigi E. Perotti, Jessica A. Martinez, Sandra M. Duarte-Vogel, Daniel B. Ennis, Holden H. Wu

**Affiliations:** 1 Department of Radiological Sciences, University of California Los Angeles, Los Angeles, CA, United States of America; 2 Department of Bioengineering, University of California Los Angeles, Los Angeles, CA, United States of America; 3 Department of Mechanical and Aerospace Engineering, University of Central Florida, Orlando, FL, United States of America; 4 Department of Radiology, Stanford University, Stanford, CA, United States of America; 5 Division of Laboratory Animal Medicine, University of California Los Angeles, Los Angeles, CA, United States of America; New York University School of Medicine, UNITED STATES

## Abstract

**Purpose:**

Real-time magnetic resonance imaging (MRI) is a promising alternative to X-ray fluoroscopy for guiding cardiovascular catheterization procedures. Major challenges, however, include the lack of guidewires that are compatible with the MRI environment, not susceptible to radiofrequency-induced heating, and reliably visualized. Preclinical evaluation of new guidewire designs has been conducted at 1.5T. Here we further evaluate the safety (device heating), device visualization, and procedural feasibility of 3T MRI-guided cardiovascular catheterization using a novel MRI-visible glass-fiber epoxy-based guidewire in phantoms and porcine models.

**Methods:**

To evaluate device safety, guidewire tip heating (GTH) was measured in phantom experiments with different combinations of catheters and guidewires. *In vivo* cardiovascular catheterization procedures were performed in both healthy (N = 5) and infarcted (N = 5) porcine models under real-time 3T MRI guidance using a glass-fiber epoxy-based guidewire. The times for each procedural step were recorded separately. Guidewire visualization was assessed by measuring the dimensions of the guidewire-induced signal void and contrast-to-noise ratio (CNR) between the guidewire tip signal void and the blood signal in real-time gradient-echo MRI (specific absorption rate [SAR] = 0.04 W/kg).

**Results:**

In the phantom experiments, GTH did not exceed 0.35°C when using the real-time gradient-echo sequence (SAR = 0.04 W/kg), demonstrating the safety of the glass-fiber epoxy-based guidewire at 3T. The catheter was successfully placed in the left ventricle (LV) under real-time MRI for all five healthy subjects and three out of five infarcted subjects. Signal void dimensions and CNR values showed consistent visualization of the glass-fiber epoxy-based guidewire in real-time MRI. The average time (minutes:seconds) for the catheterization procedure in all subjects was 4:32, although the procedure time varied depending on the subject’s specific anatomy (standard deviation = 4:41).

**Conclusions:**

Real-time 3T MRI-guided cardiovascular catheterization using a new MRI-visible glass-fiber epoxy-based guidewire is feasible in terms of visualization and guidewire navigation, and safe in terms of radiofrequency-induced guidewire tip heating.

## Introduction

Real-time magnetic resonance imaging (MRI) provides excellent soft-tissue contrast without radiation exposure [1], and is emerging as a promising alternative to X-ray fluoroscopy for intra-procedural guidance of cardiovascular catheterization [2–4] and closed chest percutaneous interventions [5]. MRI-guided catheterization has been carried out using MRI-visible markers on the catheters’ tip [6,7] or by inflating the balloon at the catheter tip by air, gadolinium [8–10], or carbon dioxide [11,12]. However, during catheterization procedures, the absence of a guidewire may result in procedure difficulties and failure [8]. Guidewires aid the navigation in complex cardiovascular regions, for example, during the catheterization of the coronary sinus [6,13]. Moreover, guidewires support the catheters and prevent their kinking. The risk for catheter kinking is especially high in procedures under real-time MRI guidance where, in general, catheters not braided with metallic wires are used to avoid radiofrequency (RF) induced heating. In this context, a novel braid-reinforced catheter with discontinuous metal wires has been recently shown [14] to preserve mechanical properties and kink resistance similar to the ones of braided catheters while limiting RF-induced heating. However, despite the possibility to use an MR safe catheter, guidewires are still needed to guide the catheter and avoid vessel damage.

Conventional guidewires are metallic and not applicable in the MRI environment due to the risk of RF-induced heating, magnetic force, and severe MR image artifacts. A guidewire used in MRI-guided procedures needs to be: 1) safe in terms of RF-induced heating; 2) reliable in terms of visualization; and 3) able to support procedural maneuvers. The lack of guidewires that satisfy these criteria poses a major challenge to real-time MRI-guided catheterization procedures [4,8].

With the goal of overcoming these challenges, commercialized non-ferromagnetic nitinol guidewires are being studied to confirm their possible use in real-time MRI-guided procedures under certain conditions such as low specific absorbing rate (SAR) sequences or low field MR scanners (3). These specific requirements limit the scope of MRI-guided catheterization. Different non-metallic designs have also been proposed for years [15–17] to overcome the low SAR, low magnetic field limitations. One type of non-metallic glass-fiber epoxy-based guidewire has been recently designed to avoid RF-induced heating and achieve reliable visualization under MRI. Preclinical evaluations of safety, visualization, and maneuverability using this glass-fiber epoxy-based guidewire have been conducted at 1.5T [17]. While 1.5T MRI scanners are the major platform for MRI-guided cardiovascular interventions, a growing amount of attention has been directed to 3T MRI scanners because of the improved signal-to-noise ratio (SNR) and clinical applications in cardiac MRI [18]. Limited evaluation of a glass-fiber epoxy-based guidewire using 3T MRI has been conducted during cryoablation of the pulmonary vein [19]. Further studies are necessary to evaluate the use of glass-fiber epoxy-based guidewires for MRI-guided cardiovascular catheterization at 3T. Therefore, the objective of this study was to evaluate the safety profile (RF-induced guidewire tip heating), visualization, and procedural feasibility of a glass-fiber epoxy-based guidewire for real-time 3T MRI-guided cardiovascular catheterization in phantoms and in healthy and infarcted porcine models.

## Methods

### Imaging facilities

**Fig 1:**
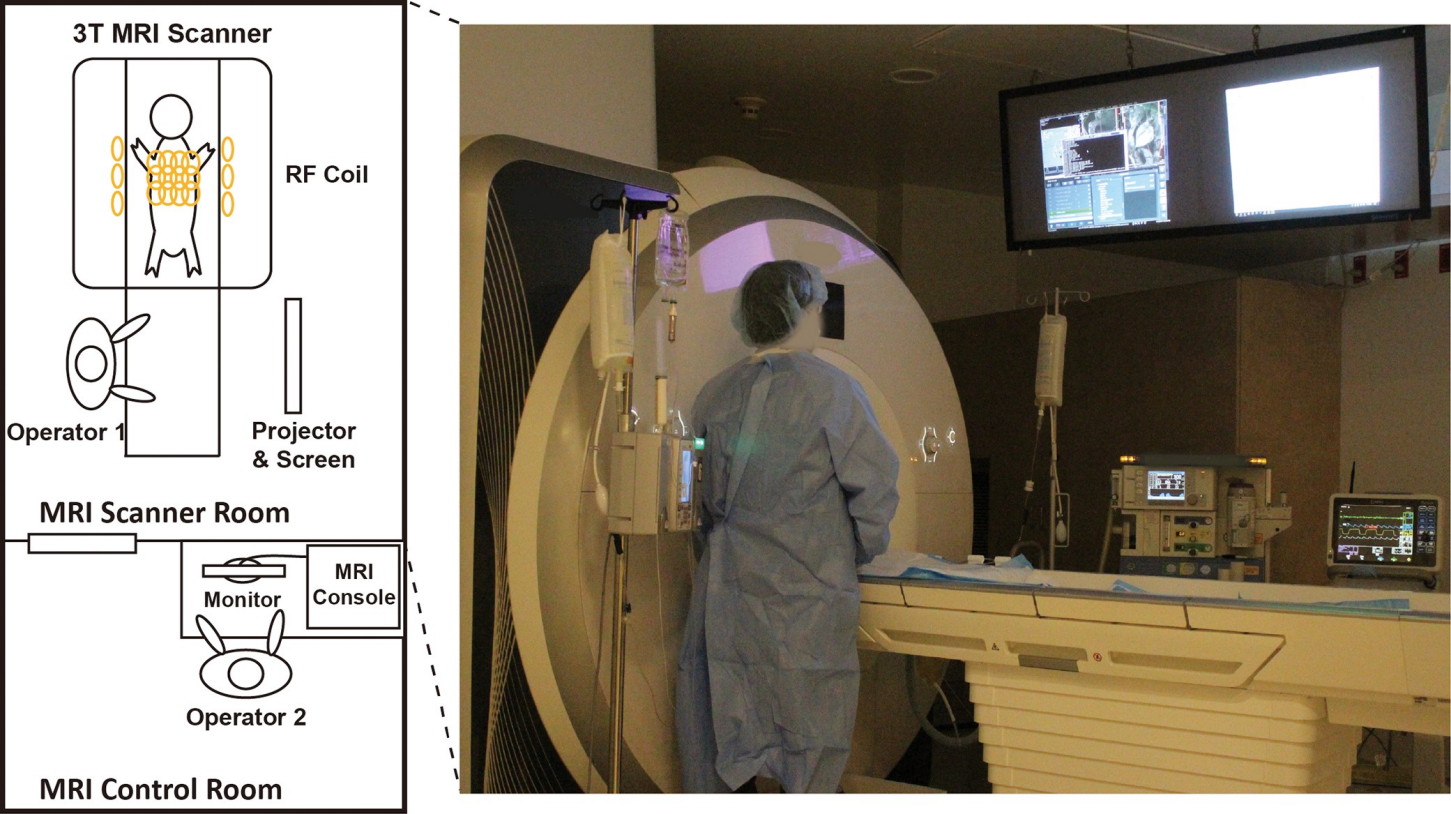
MRI scanner room setup for cardiac catheterization experiments. The guidewire maneuvering was performed by operator 1 in the MRI scanner room and the real-time scan was controlled by operator 2 in the MRI control room.

Phantom and *in vivo* porcine studies were performed in a 3T MRI scanner (MAGNETOM Prisma, Siemens Healthineers, Erlangen, Germany). The MRI suite used during *in vivo* catheterization was customized to enable intra-procedural real-time MRI guidance (**Fig 1**). Two projectors and projector screens were installed above the scanner table to display the real-time MR images and additional information such as the subject’s arterial or intraventricular pressure during the catheterization procedure to the operator in charge of navigating the guidewire under real-time MRI. An MRI compatible monitoring system (Expression, MRI Patient Monitoring System, Invivo) was used to continuously monitor the subject heart rate, peripheral capillary oxygen saturation, respiratory rate, end tidal CO_2_, invasive arterial pressure (via femoral line), body temperature, and electrocardiogram (ECG). The ECG was constantly monitored to detect premature ventricular contractions or arrhythmias due to the intraventricular guidewire and/or catheter. We will refer to the operator inside the MRI scanner room as operator 1 and to the operator in the MRI control room as operator 2. The scanner microphone system enabled communication between operators 1 and 2 for inter- and intra-procedural imaging and device setup adjustments. The two operators were not clinicians, but had extensive research experience in MRI and consulted clinicians to design and carry out the cardiovascular catheterization procedures.

### Devices: Guidewires

**Fig 2:**
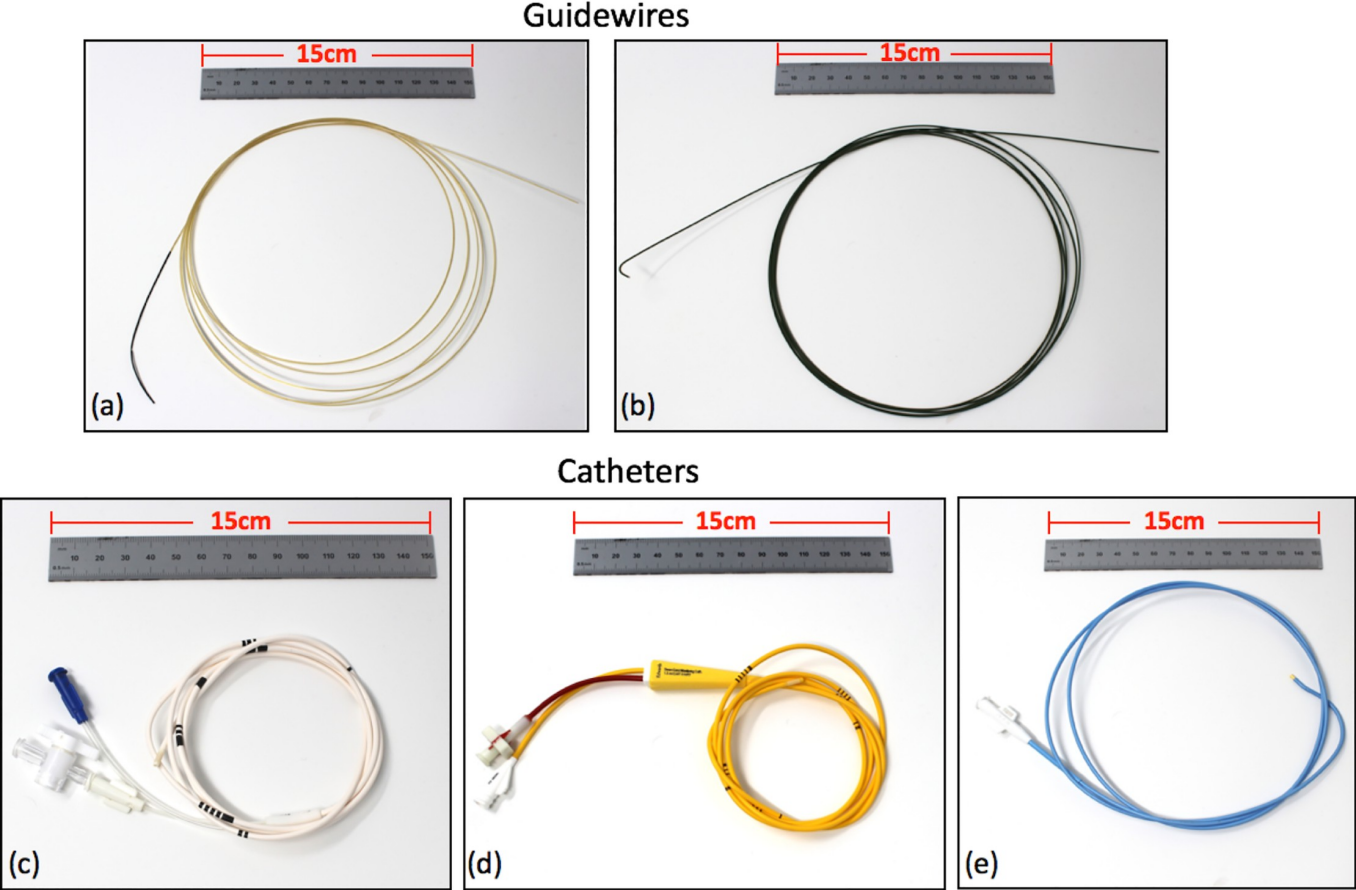
Cardiac catheterization devices evaluated in the phantom and porcine model experiments. **(a)** diameter 0.035” × length 260 cm MRI-visible glass-fiber epoxy-based guidewire (MaRVis). **(b)** 0.035” × 260 cm stainless steel guidewire (Rosen, Cook Medical) **(c)** 7 French (Fr) x 110 cm non-metallic balloon-wedge pressure catheter (Arrow, Teleflex) **(d)** 7 Fr x 110 cm non-metallic Swan-Ganz catheter (True Size Double Lumen Monitoring Catheter, Edwards Lifesciences). **(e)** 6 Fr x 100 cm braided catheter (Expo, Boston Scientific).

An MRI-visible standard stiffness glass-fiber epoxy-based guidewire was used in these experiments (MaRVis Interventional GmbH, Germany) (**Fig 2A**). The guidewire diameter and length were 0.035” and 260 cm, respectively. Each guidewire was composed of four thin rods consisting of glass or aramid fibers impregnated by epoxy resin [17]. The four rods were embedded in a polymer with one rod in the center and the other three rods at equal distance around the center one. The epoxy resin of the central rod contained iron particles along the entire length of the guidewire to generate MR susceptibility artifacts. At the distal end of the guidewire an additional MR tip marker of iron particles was positioned. The 10 cm long shapeable tip of the guidewire was very flexible and its signal void on MRI was larger than the signal void originating from the shaft marker. This allowed for reliable visualization of the guidewire tip during the procedures. The guidewire surface was covered with a hydrophilic coating.

Under fluoroscopy and in the phantom experiments, a curved stainless-steel guidewire (THSCF-35-260-1.5-ROSEN, Cook Medical) was also used (**Fig 2B**). The metal guidewire had the same diameter (0.035”) and length (260 cm) as the glass-fiber epoxy-based guidewire.

### Devices: Catheters

A 7 French (Fr) balloon wedge-pressure catheter (110 cm, Teleflex Arrow) was selected for the catheterization procedures in healthy porcine subjects (**Fig 2C**). The lumen size was sufficient to advance the fiber optic pressure transducer used to measure the intraventricular pressure once the catheter was in place. In addition, this catheter did not produce any noticeable MR image artifacts during the exams.

In infarcted porcine subjects three different catheters were used: 1) a balloon wedge-pressure catheter (7Fr x 110 cm, Teleflex Arrow) as used in the healthy subjects’ catheterization procedures; 2) a Swan-Ganz (7Fr x 110 cm, Edwards, True Size Double Lumen Monitoring Catheter) (**Fig 2D**); and 3) a braided curved catheter (6 Fr x 100 cm, Boston Scientific, Expo catheter with curve style AL2) (**Fig 2E**). The first and second catheters were chosen because they did not contain metal and did not generate any appreciable MR artifacts, while the metal braided catheter, as expected, produced noticeable MR susceptibility artifacts. The braided catheter was tested since it facilitates the navigation to the left ventricle (LV).

Although there are only a few available catheters that are MR invisible (i.e., non metal braided), different operators may choose a different catheter from the ones used in this study based on a specific procedure and personal experience.

### Porcine models

All porcine model experiments in this study were performed in strict accordance to a specific animal research protocol approved by the University of California Los Angeles Institutional Animal Care and Use Committee (protocol number: 2015–124).

The experiments described in this MRI-guided catheterization study were part of a larger overall study aimed at identifying the material properties of the passive myocardium [20]. In the overall study, intraventricular pressure was measured to provide the LV loading condition, which is critical to compute the myocardial material properties together with displacement [21] and microstructure [22] data. Healthy porcine models were used to establish a baseline for the passive myocardial stiffness while the infarcted models were used to measure regional variations in myocardial stiffness. For the purpose of the study presented here, the five infarcted subjects provided a more complex cardiovascular anatomy to evaluate the use of the guidewire for catheter navigation to the LV.

During the LV catheterization procedure to measure intraventricular pressure and for the duration of the MR exams, all subjects were under general anesthesia. Anesthesia was induced with ketamine 12.5 mg/kg and midazolam 1 mg/kg administered intramuscularly (IM). Following induction, pre-emptive analgesia was provided by administering carprofen 4 mg/kg and buprenorphine 0.02 mg/kg IM. All subjects were endotracheally intubated and anesthesia was maintained with isoflurane 1.5–2% during the imaging session. Lactated Ringer’s solution was continuously administered at 2–5 ml/kg/h during the procedure. In preparation for the catheterization procedure, access to the femoral artery was secured using the standard Seldinger technique [23], which ended by placing a 7 Fr vascular access sheath in the catheterized vessel. Patency of the catheter was confirmed by aspiration and flushing.

After the vascular access sheath had been placed, the primary LV catheterization procedure was performed under X-ray fluoroscopy by advancing a metal guidewire from the femoral artery to the LV and then a catheter over the wire. Once the catheter was in place, the wire was removed and location verified by imaging. At the end of the fluoroscopy procedure, a fiber optic pressure transducer was inserted in the catheter to measure LV intraventricular pressure. Subsequently, maintaining in place the catheter and pressure transducer, the porcine subject was transferred to the nearby MRI suite, where intraventricular pressure was constantly recorded during several MR exams aimed at measuring cardiac kinematics and microstructure. At the end of the cardiac MR exams, the catheter and pressure transducer were removed and the LV catheterization was repeated under real-time MRI guidance.

A total of ten (N = 10) swine experiments were performed in this study: five (N = 5) catheterization procedures were in healthy subjects and five (N = 5) were in subjects with a myocardial infarct. The infarct was induced by: 1) sub-selecting with a micro-guidewire (Hi-Torque, Balance Middleweight Universal Guide Wire, 0.014”, Abbot Vascular) a branch of the left anterior descending (LAD) or left circumflex (LCx) coronary artery; 2) placing a balloon catheter (MINI TREK Coronary Dilatation Catheter, 1.5 mm diameter, Abbot Vascular) just past the vessel branch point; 3) inflating the catheter balloon to avoid microsphere reflux; 4) injecting 2.5–3.0 ml of microspheres (Polybead, Polystyrene 90 micron from Polysciences Inc); 5) deflating the balloon after one minute; 6) removing the catheter from the coronary artery. During the infarct induction procedure, subjects’ ECG and vitals were constantly monitored. In one subject, the infarct was due to a thrombus formed during the LAD catheterization procedure and no microspheres were injected. The subjects with myocardial infarction underwent the MR exam six to ten weeks after the infarct induction procedure to allow for scar tissue formation and LV remodeling.

At the end of the terminal MR imaging exam, the subjects were euthanized under general anesthesia with a veterinary grade euthanasia solution (Euthasol®, Virbac. Pentobarbital 390 mg/ml and Phenytoin 50 mg/ml) administered intravenously at a volume of 1 ml/10 pounds of body weight.

### Imaging protocols for real-time MRI guidance

An interactive 2D RF-spoiled gradient echo (GRE) real-time MRI sequence was used to guide the cardiovascular catheterization procedures (BEAT_IRTTT, Siemens Healthineers, Erlangen, Germany). The imaging parameters are specified in **Table 1**. Multiple values are reported for the field of view (FOV) since it was adjusted in each experiment to accommodate the subject’s anatomy and LV/aortic arch sizes. The FOV adaptation caused slight differences (~15 ms) in the temporal resolution in each experiment.

**Table 1:** Imaging parameters.

Real-Time RF-spoiled GRE MRI Sequence at 3T			
**TR/TE**	5.8 ms– 6 ms / 3.1 ms– 3.3 ms	**Temporal Resolution**	385 ms– 400 ms
**FOV**	250 mm– 300 mm	**Flip Angle**	12°
**Voxel Size**	1.95 x 1.95 mm^2^–2.34 x 2.34 mm^2^	**Slice Thickness**	10 mm
**Parallel Imaging Factor** **/ Reference Lines**	2 / 32	**Matrix Size**	128 x 128

Real-time radiofrequency (RF) spoiled gradient echo (GRE) MRI sequence parameters at 3T. The field of view (FOV) was customized in each experiment to accommodate the subject’s anatomy and left ventricle/aortic arch sizes. TR: repetition time. TE: echo time.

Prior to beginning the LV catheterization procedure under real-time MRI, reference images were acquired using short and long axis high spatial/temporal resolution cine MRI to localize the initial imaging plane. During real-time MRI guidance, operator 2 adjusted the imaging plane to maintain the device signal void within the FOV. The interactive imaging plane control feature was essential for procedural guidance given the complex anatomy and the flexibility of the guidewire tip that makes it prone to move out of the imaging plane.

### Phantom heating experiments

Before using the glass-fiber epoxy-based guidewire for *in vivo* MRI-guided catheterization procedures, several phantom experiments were performed to evaluate guidewire tip heating (GTH) and confirm its safety at 3T. First, GTH was evaluated for the glass-fiber epoxy-based guidewire without any catheters. For comparison, a stainless-steel guidewire was also evaluated under the same conditions. Subsequently, GTH was evaluated for the glass-fiber epoxy-based guidewire while inserted in one braided catheter and two non-metallic catheters that did not exhibit any noticeable MR image artifacts.

**Fig 3:**
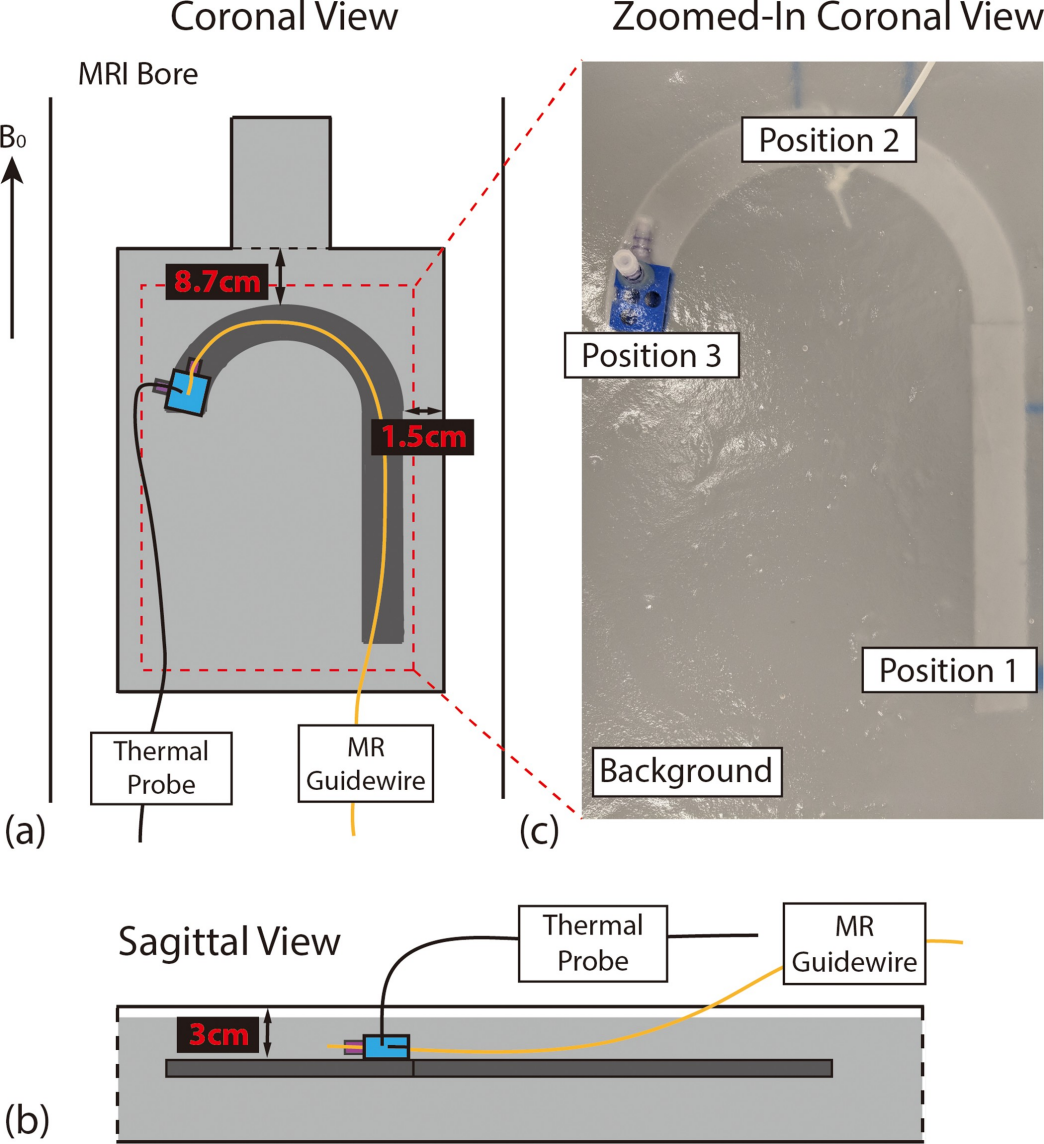
Experimental setup for evaluating device heating in the ASTM torso phantom. **(a)** Coronal and **(b)** Sagittal views of the experimental setup. **(c)** Zoomed-in view of the phantom. The MRI-visible glass-fiber epoxy-based guidewire (MR guidewire) tip and thermal probe were placed at three positions to mimic the spatial locations of the descending aorta (position 1), the aortic arch (position 2), and the left ventricle (position 3).

GTH was measured in an American Society for Testing and Materials (ASTM) torso phantom (F2182-11a) filled with a saline gel consisting of polyacrylic acid to match tissue electrical conductivity (σ = 0.47 S/m). A rail was 3D printed using polylactic acid (PLA) to support the guidewire during the phantom experiment and position its tip at three different positions simulating the locations of the descending aorta (DA), aortic arch (AA), and LV (**Fig 3**). GTH was evaluated at each of the three locations. The rail and guidewire tip were placed in the phantom and submersed in the saline gel at a depth of approximatively 3 cm. Temperature was measured with fiber optic thermal probes (Lumasense Technologies) [24]. The sensor of the temperature probe was placed in contact with, or in close proximity of, the side of the guidewire similar to the “transversal contact” configuration presented in [25]. This configuration has been shown to correspond to the highest measured temperature [25] and therefore to be the most conservative configuration. Since the temperature sensor was 2 mm away from the probe tip, this configuration corresponds to the thermal probe tip being placed about 2 mm from the guidewire tip. The accuracy and precision of the thermal probe provided by the manufacturer were ±0.5°C and 0.5°C, respectively [24]. We also analyzed the thermal probe measurements during the first 30 sec of the phantom experiments (no MR sequence applied) and found the root mean squared value of temperature change to be less than 0.1°C.

The temperature was first measured for 30 seconds at baseline without running any MRI sequences. Next, RF-induced temperature changes were measured during five minutes under normal operating mode (NOM; ≤2 W/kg whole body specific absorption rate [SAR]). The temperature changes were computed by subtracting the average temperature in the first 30 seconds from the temperature recorded during MR imaging. The following sequences were used to evaluate GTH: 1) GRE sequence with parameters adopted during *in vivo* catheterization; 2) balanced steady-state free precession (bSSFP) sequence, which is an alternative sequence for real-time MRI guidance; and 3) turbo spin echo (TSE) sequence (repetition time [TR]: 137ms; refocusing flip angle [FA]: 180°) to maximize SAR.

### Workflow for *in vivo* catheterization

After performing the MR exams to evaluate subjects’ cardiac kinematics and microstructure, the catheter placed under fluoroscopy guidance was removed leaving the access sheath in place. The catheter to be placed under real-time MRI guidance was flushed with saline and connected with a pressurized saline bag (rate of 1 ml per 10 s) using a three-way valve. Subsequently, the glass-fiber epoxy-based guidewire was flushed in the original packaging and removed from it after disconnecting the backstop. The glass-fiber epoxy-based guidewire was then inserted up to the tip of the catheter. Before proceeding, operator 1 ensured that no air bubbles were trapped in the lines, catheter, and valve.

**Fig 4:**
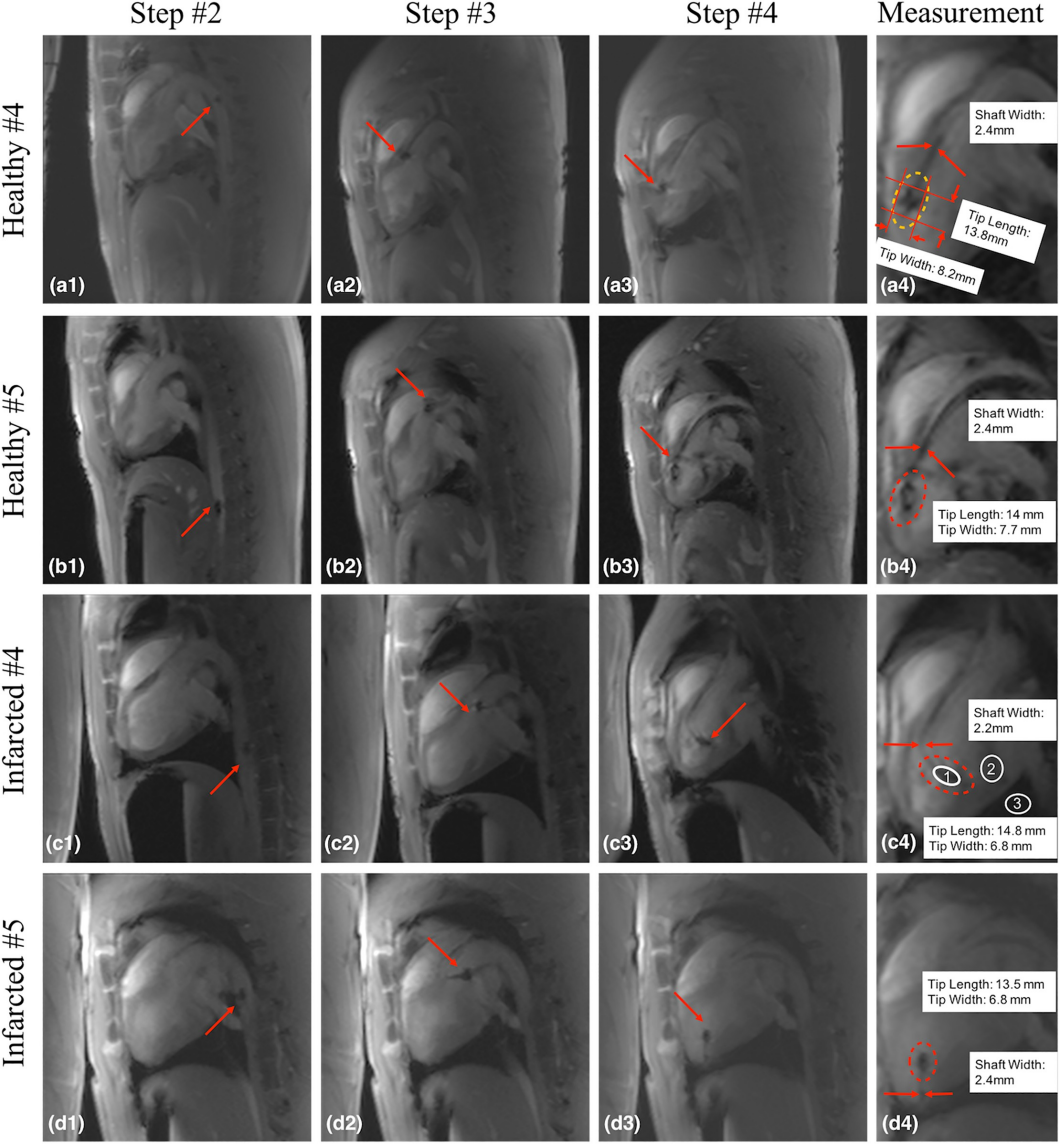
Representative examples of real-time MR images from porcine model experiments. Glass-fiber epoxy-based guidewire (MR guidewire) visualization examples during the left ventricle (LV) catheterization procedure in healthy subjects **(a, b)** and subjects with an infarct **(c, d)**. The first three columns show the MR guidewire in the descending aorta, aortic arch, and LV during steps 2–4. The corresponding signal void dimensions (e.g., **a4**) measured in the LV cavity are shown in the last column. The signal void intensity (e.g., region of interest [ROI] 1 in **c4**), surrounding blood signal intensity (e.g., ROI 2 in **c4**), and background noise (e.g., ROI 3 in **c4**) were measured to calculate the contrast-to-noise ratio (CNR) of the guidewire tip.

During the real-time MRI-guided procedure, operator 1 performed the catheterization in the MRI room using an in-room projector to display the real-time images, while operator 2 interactively adjusted the imaging plane (**Fig 1**). The entire procedure was divided into four main steps. In step 1, operator 1 introduced the guidewire and the catheter approximately 5 cm into the access sheath placed in the right femoral artery. In step 2, operator 1 pushed the guidewire into the descending aorta (**Fig 4**, a1-d1) followed by the catheter and operator 2 started interactive real-time MRI. In step 3, operator 1 steered the guidewire across the aortic arch (**Fig 4**, a2-d2) using the catheter as an aid to support and steer the guidewire tip. In step 4, operator 1 placed the catheter tip close to the aortic valve and timed the advancement of the guidewire with the cardiac rhythm to insert the guidewire into the LV (**Fig 4**, a3-d3) when the aortic valve was open. Finally, the catheter was advanced over the guidewire in the LV. The catheterization procedure was deemed successful once the catheter reached the LV. During advancement in the aorta and the LV, the glass-fiber epoxy-based guidewire always preceded the catheter tip to avoid vessel damage. ECG and subject vitals were closely monitored to detect signs of premature ventricular contraction (PVC) and arrhythmia. Real time MR images during navigation in steps 2–4 in one healthy and one infarcted subject are shown in supporting videos (**S1** and **S2** Videos).

Depending on the subject anatomy, navigation to the LV may be challenging using a non-braided flexible catheter that cannot properly support and help in steering the guidewire. In these situations, catheterization was first performed using a braided catheter that was subsequently switched on the wire for the balloon-wedge pressure catheter that does not produce noticeable MR image artifacts. The catheter exchange was achieved by: 1) advancing the guidewire in the LV; 2) slowly extracting the braided catheter while keeping the guidewire in place (the guidewire may be further advanced to maintain the guidewire tip in the LV); and 3) after removing any residual blood on the guidewire, inserting the non-braided catheter from the end of the guidewire until it reached the LV cavity. Particular attention was paid at all times to the position of the guidewire tip and to the subject’s ECG to detect any PVC and arrhythmias.

For all successful procedures, the guidewire visualization was assessed by measuring the dimensions of the signal void and the contrast-to-noise ratio (CNR) of the guidewire tip. The signal void size was measured after the guidewire tip reached the LV and was in the same location for several seconds (**Fig 4**, a4-d4). CNR was evaluated between the blood in the LV and the signal void of the guidewire tip. The time necessary to complete step 2–3 (**Fig 4**, a1-d1 to a2-d2) and step 4 (**Fig 4**, a3-d3) was also reported.

### *In vivo* heating experiments

In vivo GTH was acquired during the MRI-guided catheterization procedure in three infarcted porcine subjects (number three to five). The non-braided balloon-wedge pressure catheter was placed in the descending aorta, corresponding to the approximate position where the highest heating was detected in the phantom experiments. The glass-fiber epoxy-based guidewire and the fluoroptic thermal probe were inserted simultaneously in the catheter with the goal of maintaining the thermal probe as close as possible to the guidewire tip. GTH was measured for five minutes during imaging with the GRE and TSE sequences to evaluate the conditions during real time *in vivo* guidance and maximum SAR, respectively.

## Results

### Evaluation of device heating

#### Phantom experiments

**Fig 5:**
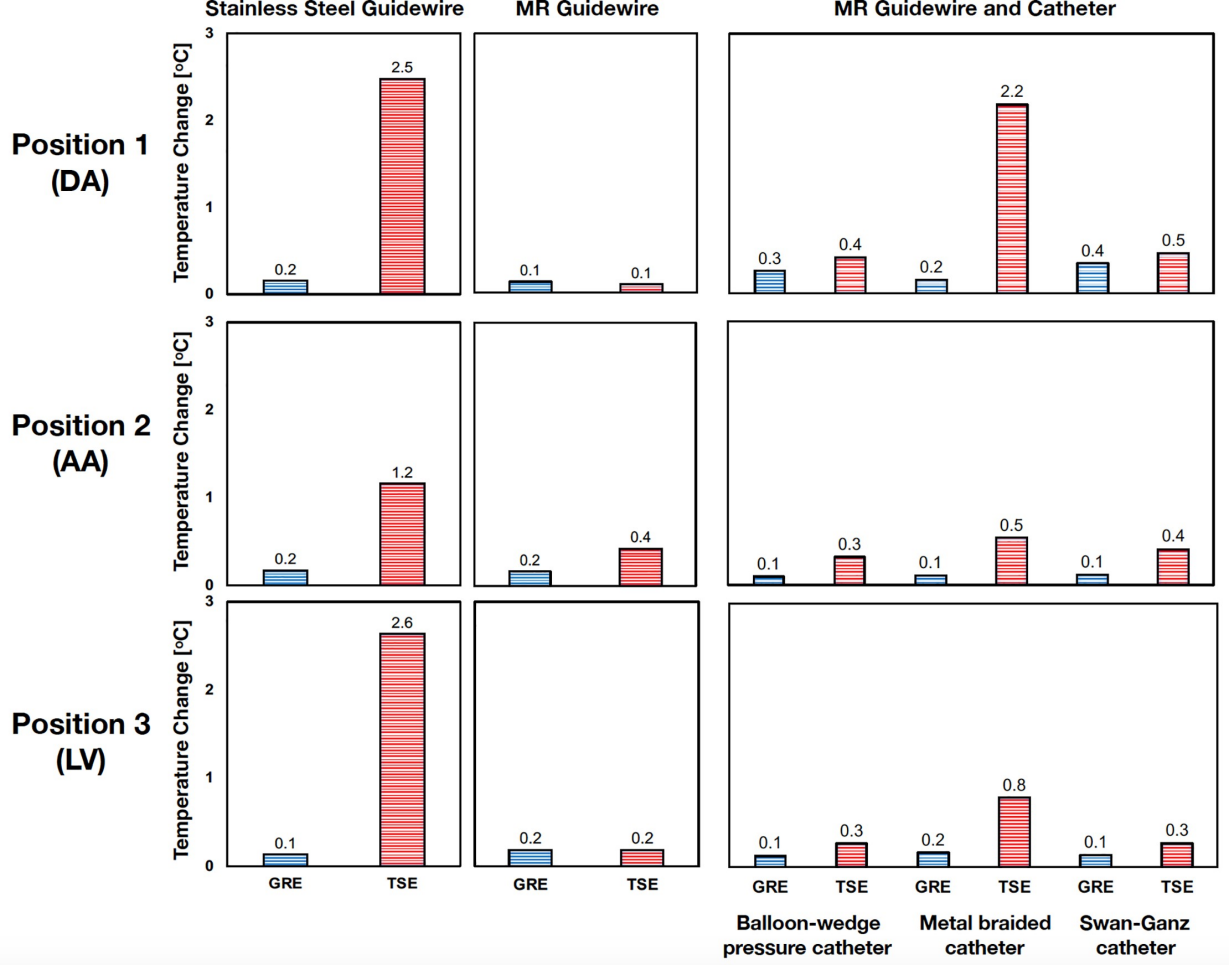
Guidewire Tip Heating (GTH) during phantom experiments. GTH measured with GRE (blue) and TSE (red) sequences during phantom experiments for a stainless-steel guidewire (left), the glass-fiber epoxy-based MR guidewire (center), and the same MR guidewire in combination with three different catheters (right). GTH was measured at three different positions representing the descending aorta (DA, position 1, top), the aortic arch (AA, position 2, middle), and the left ventricle (LV, position 3, bottom).

**Fig 6:**
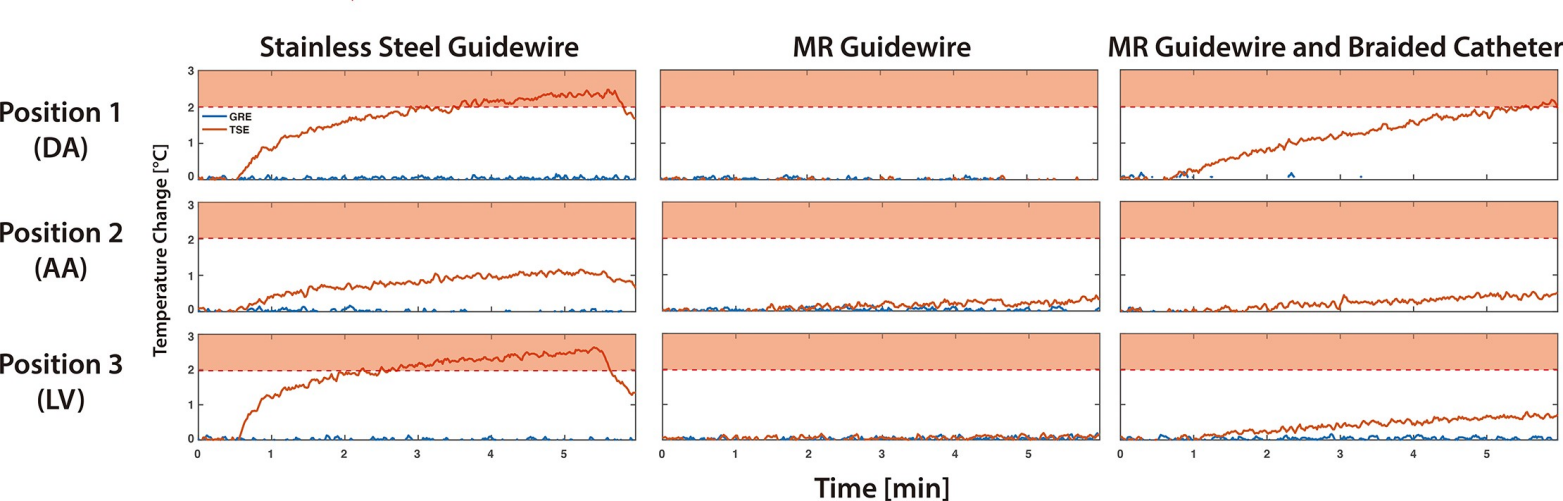
Examples of temperature recordings over time in phantom heating experiments. Guidewire tip heating (GTH) for stainless steel guidewire only, glass-fiber epoxy-based guidewire only, and glass-fiber epoxy-based guidewire with braided catheter over time during the phantom heating experiments using the gradient echo (GRE, blue line) and turbo spin echo (TSE, red line) sequences. The guidewire tip was placed at position 1 to mimic the descending aorta (DA) location, position 2 to mimic the aortic arch (AA) location, and position 3 to mimic the left ventricle (LV) location. A clear increase in temperature is detected at the tip of the stainless-steel guidewire at positions 1 and 3 during imaging with the TSE sequence. The red zone represents a GTH greater than 2°C, which is not considered procedurally safe. Note that only positive temperature changes above baseline are shown since they correspond to the RF-induced heating.

GTH was dependent on the type of guidewire, its tip location, and the type of MRI sequence used (**Table 2** and **Fig 5**). Representative examples of temperature recordings over time are shown in **Fig 6.** These temperature recordings are also available as **S1 Datafile**. During imaging using the GRE sequence, whole-body SAR reported by the scanner was 0.04 W/kg and GTH was less than or equal to 0.19°C for both the glass-fiber epoxy-based and the stainless-steel guidewires when used without a catheter. During imaging using the TSE sequence, whole-body SAR reported by the scanner was 1.88 W/kg. Maximum GTH was observed for the stainless-steel guidewire at positions 1 and 3 corresponding to the DA and LV locations (~2.5°C). Maximum GTH for the glass-fiber epoxy-based guidewire was 0.43°C and observed at position 2, corresponding to the AA location.

**Table 2:** Phantom heating evaluation results.

**a. MR guidewire**	**Maximum ΔT [°C]** **GRE**	**Maximum ΔT [°C]** **bSSFP**	**Maximum ΔT [°C]** **TSE**
Position 1 (DA)	0.13	0.11	0.10
Position 2 (AA)	0.17	0.43	0.43
Position 3 (LV)	0.19	0.29	0.19
**b. Stainless steel guidewire**	**Maximum ΔT [°C]** **GRE**	**Maximum ΔT [°C]** **bSSFP**	**Maximum ΔT [°C]** **TSE**
Position 1 (DA)	0.16	2.16	2.47
Position 2 (AA)	0.17	0.12	1.16
Position 3 (LV)	0.13	0.16	2.63
**c. MR guidewire and balloon-wedge pressure catheter**	**Maximum ΔT [°C]** **GRE**	**Maximum ΔT [°C]** **bSSFP**	**Maximum ΔT [°C]** **TSE**
Position 1 (DA)	0.26	0.49	0.42
Position 2 (AA)	0.10	0.19	0.32
Position 3 (LV)	0.13	0.32	0.27
**d. MR guidewire and braided catheter**	**Maximum ΔT [°C]** **GRE**	**Maximum ΔT [°C]** **bSSFP**	**Maximum ΔT [°C]** **TSE**
Position 1 (DA)	0.16	0.17	2.18
Position 2 (AA)	0.11	0.64	0.54
Position 3 (LV)	0.16	1.15	0.79
**e. MR guidewire and Swan-Ganz catheter**	**Maximum ΔT [°C]** **GRE**	**Maximum ΔT [°C]** **bSSFP**	**Maximum ΔT [°C]** **TSE**
Position 1 (DA)	0.35	0.54	0.47
Position 2 (AA)	0.12	0.31	0.41
Position 3 (LV)	0.14	0.26	0.28

Maximum increase in temperature (ΔT) at the guidewire tip during the phantom heating experiments at 3T. Results are shown for the MRI-visible glass-fiber epoxy-based guidewire (MR guidewire) and stainless steel guidewire **(a**, **b)** and for three different catheters together with the MR guidewire **(c-e)**. The temperatures were measured with the guidewire tip at three positions to mimic the spatial locations for the descending aorta (DA, position 1), the aortic arch (AA, position 2), and the left ventricle (LV, position 3). GRE: gradient echo. bSSFP: balanced steady-state free precession. TSE: turbo spin echo.

The glass-fiber epoxy-based guidewire was also tested in combination with the balloon-wedge pressure catheter, the Swan-Ganz catheter, and the braided catheter. GTH did not exceed 0.54°C when the glass-fiber epoxy-based guidewire was used in combination with the balloon-wedge pressure and Swan-Ganz catheters under any sequence. GTH reached 2.18°C when the glass-fiber epoxy-based guidewire was used in combination with the braided catheter during imaging with TSE at position 1 (corresponding to the DA).

#### *In vivo* experiments

GTH measured in three *in vivo* porcine experiments using the GRE and TSE sequences was less than 0.3°C for the glass-fiber epoxy-based guidewire combined with the balloon-wedge pressure catheter (**Table 3**).

**Table 3:** *In vivo* heating evaluation results.

*In vivo* Experiment using MR guidewire	Maximum ΔT [°C] GRE	Maximum ΔT [°C] TSE
**Experiment 1**	0.17	0.15
**Experiment 2**	0.16	0.20
**Experiment 3**	0.26	0.25

The maximum temperature change (ΔT) close to the glass-fiber epoxy-based MR guidewire tip was measured *in vivo* at the end of the catheterization procedure in three infarcted porcine models. GRE: gradient echo. TSE: turbo spin echo.

### Evaluation of procedural feasibility and guidewire visualization

The catheter was successfully placed in the LV in all five healthy porcine subjects using the glass-fiber epoxy-based guidewire under real-time MRI guidance. The catheterization procedure was not successful in the first two infarct subjects due to a complex anatomy and a learning curve, and was successful in the three subsequent infarct subjects. If unsuccessful, the catheterization trial was interrupted approximatively 30 minutes after starting the procedure. The guidewire signal void size and CNR between the guidewire tip signal void and blood in the LV are reported for all successful cases in Tables **4** and **5**.

**Table 4:** Healthy porcine model experimental results.

Healthy Porcine Model Experiments	Procedure Time [min:sec]		CNR (blood-signal void) at 3 positions			Signal Void Size (mm)		
	Step 2–3	Step 4	DA	AA	LV	TL	TW	SW
**Experiment 1**	10:59	2:12	72.4	93.1	117.3	12.1	5.0	2.3
**Experiment 2**	1:00	0:12	31.5	37.8	37.6	11.5	5.7	2
**Experiment 3**	2:05	0:49	39	96.0	74.4	13.6	6.2	2.1
**Experiment 4**	0:20	4:49	34.2	55.5	45.2	13.8	8.2	2.4
**Experiment 5**	13:43	1:27	33.0	47.0	48.5	14	7.7	2.2

Real-time MR images acquired with a gradient echo (GRE) sequence in five healthy porcine model experiments were analyzed to compute: a) procedure times for step 2–3 and step 4; b) contrast-to-noise ratio (CNR) for guidewire visualization in three positions including the descending aorta (DA), aortic arch (AA), and left ventricle (LV); and c) the MRI signal void size of the glass-fiber epoxy-based guidewire in terms of tip length (TL), tip width (TW), and shaft width (SW).

**Table 5:** Infarct porcine model experimental results.

Infarcted Porcine Model Experiments	Procedure Time [min:sec]		CNR (blood-signal void) at 3 positions			Signal Void Size (mm)		
	Step 2–3	Step 4	DA	AA	LV	TL	TW	SW
**Experiment 3**								
**Trial 1 (MR guidewire + Braided catheter -> balloon-wedge pressure catheter)**	4:14	0:24	32.6	46.7	54.5	12.1	5	2.3
	switch	1:14						
**Experiment 4**								
**Trial 1 (MR guidewire + Swan-Ganz catheter)**	3:41	3:03	27.5	41.2	37.6	14.8	6.8	2
**Trial 2 (MR guidewire + Swan-Ganz catheter)**	0:11	0:16	31.7	35.8	36.6	12.1	6.4	2
**Trial 3 (MR guidewire +** **balloon-wedge pressure catheter)**	1:45	0:05	33.6	41.2	40.9	12.5	6.2	2.1
**Trial 4 (MR guidewire +** **balloon-wedge pressure catheter)**	1:09	0:07	31.5	38.4	39.3	12.2	5.9	2.1
**Experiment 5**								
**Trial 1 (MR guidewire +** **balloon wedge pressure catheter)**	3:24	0:23	47.6	64.2	74.4	13.5	6.8	2.4
**Trial 2 (MR guidewire + Swan-Ganz catheter)**	0:42	1:00	61.3	66.5	62.8	12.2	6	2.1
**Trial 3 (MR guidewire +** **balloon-wedge pressure catheter)**	0:42	0:17	54.3	78.2	75.5	12.8	6.2	2

Real-time MR images acquired during three successful infarcted porcine model experiments were analyzed to compute: a) procedure times for step 2–3 and step 4; b) contrast-to-noise ratio (CNR) for the glass-fiber epoxy-based MR guidewire visualization in three positions including the descending aorta (DA), aortic arch (AA), and left ventricle (LV); and c) MRI signal void size of the guidewire in terms of tip length (TL), tip width (TW), and shaft width (SW). Multiple catheterization trials were performed in experiments 4 and 5. During experiment 3, the aortic arch was first reached using a metal braided catheter (Expo, Boston Scientific) that was then switched on the wire to a balloon-wedge pressure non-metallic catheter (Arrow, Teleflex).

In healthy porcine subjects, step 2–3 and step 4 of the catheterization procedure were only performed once. Depending on the subject’s cardiovascular anatomy, the shortest (longest) time in min:sec was 0:20 (13:43) for step 2–3 and 0:12 (4:49) for step 4. The average time for the entire catheterization procedure (steps 2 to 4) in healthy subjects was 7:31 (standard deviation 6:16). A complete list of the procedure times in healthy subjects is reported in **Table 4**.

Multiple catheterization trials were performed in each successful MRI-guided catheterization experiment in the infarcted porcine subjects. During each experiment several catheters were tested either by switching the catheter on the glass-fiber epoxy-based guidewire or by repeating the procedure after removing both the guidewire and the catheter. The catheters used and the times for step 2–3 and 4 are reported in **Table 5.** The average time for the entire catheterization procedure (steps 2 to 4) in all successful infarcted studies was 2:40 (standard deviation 2:10). The average time for the entire catheterization procedure (steps 2 to 4) in all successful healthy and infarcted subjects was 4:32 (standard deviation 4:41).

During experiments in the fourth infarcted porcine model, we tested two additional catheters identical to the balloon-wedge pressure and Swan-Ganz catheters previously described, except that their tip and curved region close to the tip were marked with MRI-visible iron particles (similar to the tip markers of the glass-fiber epoxy-based guidewire; provided by MaRVis Interventional GmbH). A catheterization procedure using a catheter with MRI-visible markers is shown in a supporting video (**S2 Video**). For the catheters without MRI-visible markers or signal void due to a braided tip, operator 1 confirmed the final position of the catheter in the LV by injecting Gadolinium-based MR contrast agent (volume <10 ml) through the catheter.

## Discussion

Our studies were motivated by the lack of a comprehensive evaluation of the novel glass-fiber epoxy-based MR guidewire in 3T MRI scanners. The study design of the porcine model experiments followed a clinically relevant workflow for LV catheterization. The phantom experiments were performed according to the standard ASTM test method for evaluating RF-induced heating in MRI [26]. The major findings of this study from the phantom heating experiments and *in vivo* porcine catheterization experiments showed minimal RF-induced device heating, consistent visualization, and procedural feasibility for using a novel glass-fiber epoxy-based guidewire in a 3T MRI scanner.

### Safety with respect to RF-induced heating

The absence of notable RF-induced heating for the glass-fiber epoxy-based guidewire was shown during the phantom studies and confirmed with *in vivo* measurements. Minimal GTH was also shown when the guidewire was combined with different catheters that are not visible under MRI. GTH greater than 2°C was only observed when the glass-fiber epoxy-based guidewire was utilized with a metal braided catheter and using a high SAR TSE sequence. However, metal braided catheters are not an optimal choice since they induce a large MR image artifact that compromises the image quality. Metal braided catheters may be used only in the initial phase of the catheterization procedure without a high SAR sequence to aid guidance in specific regions, e.g., in passing the aortic arch. Safe use of the glass-fiber epoxy-based guidewire with a non-metallic catheter was also shown *in vivo* with both GRE and TSE sequences (GTH <0.3 C°). The low GTH is due to the construction of the glass-fiber epoxy-based guidewire in which metal particles do not form a continuous conductive medium. In previous work [14] it has been shown that RF-induced heating can be significantly decreased or eliminated if the length of each metal segment in a guidewire is less than a quarter wave length of a given electromagnetic field. The iron particles embedded in the glass-fiber epoxy-based guidewire adopted in this study are micrometer particles and they are separated from each other. Therefore, the glass-fiber epoxy-based guidewire has very low electrical conductivity and consequently no RF-induced heating can be expected. This has been confirmed in our phantom and *in vivo* testing.

The glass-fiber epoxy-based guidewire was previously tested using bSFFP at 1.5T [17]. The present study extends the evaluation to 3T and with high SAR bSFFP and TSE sequences. Therefore, further sequence development for MRI-guided catheterization can be pursued at 3T and the use of sequences with higher SAR can be explored as well.

Different levels of temperature change were recorded in different positions due to the inhomogeneity of the electric (E)-field as well as its orientation with respect to the guidewire and catheters [27,28]. First, greater E-field magnitude is observed close to position 1, whereas a lower E-field magnitude is found at the center of the phantom. Temperature increase is proportional to the magnitude of the E-field. Secondly, only the tangential component of the E-field will induce currents along the guidewire. In position 1, the E-field lines are aligned with the guidewire/catheter while in position 2 they are mostly perpendicular to it. Hence, less heating is expected in position 2 regardless of the amount of power applied by the RF transmit coil. Lastly, the E-field is more homogenous toward the scanner isocenter (closer to position 3) and a more homogenous E-field would excite the guidewire more effectively and result in greater heating. The interplay of these factors combined with the applied MR sequences led to different levels of heating in positions 1, 2, and 3 for the guidewires and catheters we tested.

### Visualization

The visualization of the glass-fiber epoxy-based guidewire with a real-time RF spoiled GRE sequence was sufficient to support the catheterization procedure. In the LV, CNR between the blood and the guidewire signal void was sufficient (CNR: 57.5±28). Despite the noticeable variation in CNR values, the guidewire tip and shaft were consistently visualized with limited variations across all procedures: tip length: 12.5±1.1 mm, tip width: 6.4±1.3 mm, shaft width: 2±0.2 mm. The difference in the width of the signal void between the guidewire shaft and tip was instrumental to determine the tip location. In contrast to only providing support for the catheter as presented in [19], in this study we used the glass-fiber epoxy-based guidewire to provide direct guidance for the procedure through passive visualization.

Fast advancement of the guidewire could lead to blurred signal void on dynamic images and hinder guidewire tracking. However, adjustment of the image plane and reducing the speed of guidewire motion, especially at key anatomical locations such as the aortic arch and valve, allowed precise localization of the guidewire tip. In order to provide dynamic guidance with improved imaging temporal resolution (e.g., 10 frames per second), different MRI methods such as radial sampling [29,30] can be used.

Compared with the guidewire tip signal void dimensions at 1.5T using the bSSFP sequence [17], the signal void dimensions at 3T using a GRE sequence are slightly larger. This may limit the ability to effectively direct the guidewire in complex vascular structures and small anatomical regions. Nevertheless, the visualization of the glass-fiber epoxy-based guidewire tip and shaft in the normal LV and aorta adequately supported MRI-guided catheterization.

### Procedural feasibility

The catheterization procedure times were within a reasonable range and demonstrated the feasibility of the LV catheterization procedure in both healthy and infarcted porcine models. The catheterization procedures in the first two infarcted subjects were not successful due to a more complex vascular and cardiac anatomy that rendered the selection of the image plane to visualize the full aortic arch and passive guidewire signal void more challenging. The lack of an appropriate catheter to support the guidewire further increased the difficulty of performing the catheterization procedure in the first two infarcted subjects. Catheterization in the three subsequent infarcted subjects were all successful due to increased operator experience and either: 1) using a braided catheter and switching it on the wire for an MRI-invisible catheter after reaching the LV; or 2) using a catheter with MRI-visible markers close to its tip to visualize its exact position under MRI. Both of these strategies could be considered in presence of a more complex anatomy. Using the first strategy, operator 1 was able to better shape the tip of the catheter and turn the catheter to direct the wire in the desired direction. The second strategy of using MRI-visible markers on the catheters allowed operator 1 to better direct the wire by knowing the exact position of the catheter tip with respect to the guidewire tip. If the catheter remains in place, e.g., to record intraventricular pressure, the presence of MRI-visible markers may compromise the image quality during the MR exam and switching the catheters on the wire may be preferred. However, the MRI-visible markers on the catheters at this stage were experimental and can be improved and optimized for a specific application.

The longest time in step 2–3 (**Table 4**) corresponded to the case where the guidewire continuously reached the carotid artery instead of turning into the aortic arch. The longest time recorded in step 4 was due to the folding of the guidewire in the aorta and additional time was required to unfold the guidewire before continuing the procedure. Both events were special cases and the increase in procedural time was independent from the guidewire visualization. Note that after this learning process, the three final experiments in infarcted subjects (**Table 5**) were all completed with short times for steps 2–3 and 4.

### Limitations and future work

The bSSFP sequence is a widely used real-time MRI sequence for cardiac interventions, especially at 1.5T. Being a high SAR sequence, bSSFP has an increased potential to cause RF-induced device heating at 3T and therefore could benefit from being combined with a glass-fiber epoxy-based guidewire. However, in our study at 3T, large flow and banding artifacts appeared using the bSSFP sequence due to B_0_ inhomogeneity when maneuvering the guidewire in both phantom and porcine models [31]. Due to this reason, we opted to use the spoiled GRE sequence, which is less sensitive to the off-resonance effects caused by B_0_ field inhomogeneity [32]. Furthermore, the GRE sequence led to consistently low RF-induced GTH at all locations and for all combinations of guidewires and catheters in the phantom experiments. In future studies, the experimental setup should be optimized (e.g., including higher-order shimming) to limit artifacts for real-time bSSFP at 3T. The minimal glass-fiber epoxy-based guidewire tip heating observed at 3T encourages the use and design of bSSFP and other MRI sequences for real-time guidance at this higher magnetic field strength.

Although the glass-fiber epoxy-based guidewire can be safely employed in real-time MRI applications in contrast to the tested metal guidewire, its bending flexibility, torque, and maneuverability are not the same as the ones of a metal guidewire, whose structural properties cannot be reproduced at this time using a multi-composite guidewire. The glass-fiber epoxy-based MR guidewire tended to rest against the vessel wall around sharp turns in the vasculature. On the other hand, the metal guidewire was easier to advance, twist and direct. However, the increased soft-tissue contrast under real-time MRI and the absence of ionizing radiation, may, depending on the procedure, be of greater importance than the higher maneuverability granted at present by using a metal guidewire. It is important to notice that guidewire maneuverability is also a function of the operator preferences and experience, and further evaluation by trained clinicians is needed. Additionally, guidewire maneuverability is affected by the interaction between the guidewire and the chosen catheter. The ability to increase soft tissue contrast and visualization by performing the procedure under MR guidance may decrease the complexity of procedural steps and thereby decrease the mechanical and maneuverability requirements on the guidewire.

## Conclusions

This pre-clinical study provided evidence for the safety, consistent MRI visualization, and feasibility of using an MRI-visible glass-fiber epoxy-based guidewire for real-time 3T MRI-guided cardiovascular catheterization procedures. The favorable characteristics of this glass-fiber epoxy-based guidewire may allow a range of catheterization procedures that take advantage of the increased soft-tissue contrast and absence of ionizing radiation provided by real-time 3T MRI guidance. Further investigations are necessary to translate and evaluate this new strategy in clinical applications.

## Supporting information

S1 Datafile Guidewire tip heating (GTH) for stainless steel guidewire only, glass-fiber epoxy-based guidewire only, and glass-fiber epoxy-based guidewire with a braided catheter over time during the phantom heating experiments using the gradient echo (GRE) and turbo spin echo (TSE) sequences. These temperature recordings correspond to Fig 6 in the manuscript.
(ZIP)

S1 Video Video of a Catheterization Procedure using a Glass-Fiber Epoxy-Based Guidewire in a Heathy Porcine Model. (MP4)

S2 Video Video of a Catheterization Procedure using a Glass-Fiber Epoxy-Based Guidewire in a Porcine Model with an Infarct. The catheter used in the procedure has three MRI visible markers in the section close to the tip.
(MP4)
